# Engineering Smut Resistance in Maize by Site-Directed Mutagenesis of *LIPOXYGENASE 3*

**DOI:** 10.3389/fpls.2020.543895

**Published:** 2020-10-21

**Authors:** Krishna Mohan Pathi, Philipp Rink, Nagaveni Budhagatapalli, Ruben Betz, Indira Saado, Stefan Hiekel, Martin Becker, Armin Djamei, Jochen Kumlehn

**Affiliations:** ^1^Plant Reproductive Biology, Leibniz Institute of Plant Genetics and Crop Plant Research (IPK), Gatersleben, Germany; ^2^Biotrophy & Immunity, Leibniz Institute of Plant Genetics and Crop Plant Research (IPK), Gatersleben, Germany

**Keywords:** Cas9, corn, CRISPR, genome editing, guide RNA, targeted mutagenesis, susceptibility factor, *Ustilago maydis*

## Abstract

Biotic stresses caused by microbial pathogens impair crop yield and quality if not restricted by expensive and often ecologically problematic pesticides. For a sustainable agriculture of tomorrow, breeding or engineering of pathogen-resistant crop varieties is therefore a major cornerstone. Maize is one of the four most important cereal crops in the world. The biotrophic fungal pathogen *Ustilago maydis* causes galls on all aerial parts of the maize plant. Biotrophic pathogens like *U. maydis* co-evolved with their host plant and depend during their life cycle on successful manipulation of the host’s cellular machinery. Therefore, removing or altering plant susceptibility genes is an effective and usually durable way to obtain resistance in plants. Transcriptional time course experiments in *U. maydis*-infected maize revealed numerous maize genes being upregulated upon establishment of biotrophy. Among these genes is the maize *LIPOXYGENASE 3* (*LOX3*) previously shown to be a susceptibility factor for other fungal genera as well. Aiming to engineer durable resistance in maize against *U. maydis* and possibly other pathogens, we took a Cas endonuclease technology approach to generate loss of function mutations in *LOX3. lox3* maize mutant plants react with an enhanced PAMP-triggered ROS burst implicating an enhanced defense response. Based on visual assessment of disease symptoms and quantification of relative fungal biomass, homozygous *lox3* mutant plants exposed to *U. maydis* show significantly decreased susceptibility. *U. maydis* infection assays using a transposon mutant *lox3* maize line further substantiated that *LOX3* is a susceptibility factor for this important maize pathogen.

## Introduction

Maize (*Zea mays* L.) is one of the most important cereal crops in the world. As a fast growing C4 plant, its kernels are used for direct human consumption, its biomass for feed and biofuel production, and it is also a source of raw material for the chemical and food industries (Pathi et al., 2013). Crop diseases have been leading to significant reductions in both crop yield and product quality and this can threaten global food security (Pegoraro et al., 2011). On a worldwide scale, annual losses in maize caused by pathogens account for approximately 75 million metric tons^1^ despite the application of pesticides that are costly and can have detrimental effects on the environment and biodiversity. In the context of a growing world population and food demand, there is an urgent requirement to develop crop varieties with broad-spectrum resistance (Dangl et al., 2013). Over evolutionary times, plants co-evolved with the selection pressure of invading pathogens, leading to a sophisticated, multilayered, and interconnected innate immune system. As a first layer of self–nonself recognition, plants detect pathogen-associated molecular patterns (PAMPs) or danger signals like damage-associated molecular patterns (DAMPs) via cell surface-localized pattern recognition receptors (PRRs). Upon extracellular ligand binding, intracellular signaling events stimulate the production of reactive oxygen species (ROS) in the extracellular space and intracellularly a transcriptional reprogramming of the plant. This reaction is called PAMP-triggered immunity (PTI) which strengthens defense, for example, by the secretion of antimicrobial peptides/compounds (Saijo et al., 2018). Successful pathogens evolved secreted molecules, so-called effectors, that suppress PTI responses on various levels, enabling the pathogen to establish on its host despite its initial recognition (Uhse and Djamei, 2018). Whereas some effectors directly target host immunity components to block their activity, others act rather indirectly to subvert the host defense system. Often, build-in growth-defense antagonisms in the plant metabolism and hormone signaling are exploited to suppress specific defense pathways depending on the lifestyle and the specific requirements of the pathogen.

Whereas necrotrophic pathogens kill the host cells to obtain the nutrients contained therein, hemibiotrophic pathogens establish an essential phase of interaction with host cells, which is later followed by necrotrophy. Biotrophs depend on a prolonged and intensified interaction with the manipulated, living host, which, however, usually restricts their host range (Uhse and Djamei, 2018). Depending on the lifestyle of the attacker, plants have evolved adapted strategies systemically coordinated by specific defense phytohormones to respond and efficiently restrict the invader. Generally spoken, whereas biotrophs are fought off by local cell death in a hypersensitive response and mainly via salicylic acid-regulated defenses, necrotrophs are restricted by the ethylene (ET) and jasmonic acid (JA) signaling-coordinated production of phytoalexins but also by secreted proteases and other counterattacking enzymes. These mainly antagonistically acting phytohormones (JA/ET vs. SA) are further partially antagonistic or synergistically interconnected with growth hormones like indole-3-acetic acid (IAA), cytokinin (CK), or gibberellin (GA) which are important modulators of immune signaling in plants (Kazan and Lyons, 2014). These interconnections are exploited by co-evolving pathogens to suppress host responses that are non-favorable for themselves.

In rice, immunity against the hemibiotrophic fungus *Magnaporthe oryzae* is conferred by JA-mediated defense (Riemann et al., 2013). *M. oryzae* secretes an antibiotic biosynthesis monooxygenase that converts both fungal and host-derived JA to 12OH-JA, thereby impeding JA-mediated immunity (Patkar et al., 2015). By contrast, the necrotrophic grapevine pathogen *Lasiodiplodia mediterranea* activates JA signaling through the production of the JA ester lasiojasmonate A (LasA). LasA can be converted to JA-Ile, a robust mediator of JA signaling and inducer of cell death. LasA is therefore proposed to act as a metabolite effector in late stages of infection that activates JA-mediated cell death and facilitates necrotrophy (Chini et al., 2018). The hemibiotrophic pathogen *Phytophthora sojae* suppresses ET biosynthesis by secreting the polymorphic RxLR effector PsAvh238 that itself facilitates the infection process (Yang et al., 2019). PsAvh238 interacts with and destabilizes Type 2 1-aminocyclopropane-1-carboxylate synthases (ACS) of soybean (Yang et al., 2019). ET production is directly related to ACS activity (Christians et al., 2009; Skottke et al., 2011; Li et al., 2012; Helliwell et al., 2016). The necrotrophic fungal pathogen *Cochliobolus miyabeanus* requires ET signaling for pathogenesis. It pursues a different infection strategy in that it mimics infected tissues by producing ET. Colonization of *C. miyabeanus* is significantly compromised by blocking ET biosynthesis by means of chemical inhibitors (Van Bockhaven et al., 2015). *Verticillium dahliae* and *P. sojae* reduce the SA defense hormone biosynthesis by secreting the isochorismatase VdIsc1 and PsIsc1 effectors, respectively (Liu et al., 2014). These enzymes are thought to inhibit the biosynthesis of SA by converting the precursor isochorismate to 2,3-dihydro-2,3-dihydroxybenzoate and pyruvate (Liu et al., 2014). *Pseudomonas syringae* produces phytotoxin coronatine (COR), a toxin that mimics the plant hormone JA, which enhances bacterial growth as well as the development of disease symptoms and promotes systemic susceptibility (Mittal and Davis, 1995; Brooks et al., 2005; Cui et al., 2005).

One of the best-studied biotrophic pathogens is the smut fungus *Ustilago maydis* which causes galls on all aerial parts of its host plants maize (*Zea mays*) and teosinte (*Euchlaena mexicana*). *U. maydis* typically infects 1 to 5 % of the plants within commercial maize fields (Christensen, 1963; Shurtleff, 1980) thereby lowering yield and quality of the crop (Immer and Christensen, 1928; Billett and Burnett, 1978). To establish a biotrophic interaction with its host plant, *U. maydis* secretes likely hundreds of effectors to suppress immunity and to redirect the host metabolism. Among the functionally characterized effectors are those involved in PTI suppression such as the peroxidase inhibitor Pep1 (Hemetsberger et al., 2012), the cysteine protease inhibitor Pit2 (Mueller et al., 2013) and the recently identified Pleiades that are represented by ten clustered effectors involved in suppression of PAMP-triggered ROS burst (Navarrete et al., 2019). Other effectors like the secreted chorismate mutase (Cmu1) converts chorismate to prephenate, by which a substrate for SA biosynthesis is removed (Djamei et al., 2011). Besides SA signaling, *U. maydis* has been shown to actively manipulate JA/ET signaling in maize by secretion of the Jsi1 effector that targets the c-terminal domain of TOPLESS (Darino et al., 2019). Furthermore, *U. maydis* directly produces auxin during infection (Reineke et al., 2008) and has also been shown to generate cytokinins, which indicates that it uses various phytohormone signaling pathways to manipulate its host (Morrison et al., 2015).

The importance of fungal induction of jasmonate and auxin signaling during establishing biotrophy lies in their antagonistic effect on PTI and SA-related defense responses (Hilbert et al., 2013; Naseem et al., 2015; Zhang L. et al., 2017). In agreement with this principle, an extensive transcriptional and metabolic profiling study revealed that auxin- and JA-regulated genes are upregulated upon *U. maydis* infection (Doehlemann et al., 2008). Production of JA in maize was shown to be regulated by 9-oxylipins (Borrego and Kolomiets, 2016). Upon *U. maydis* infection, *LOX3* is among the transcriptionally upregulated maize genes. Intriguingly, *LOX*3 was demonstrated to be a susceptibility factor for *Fusarium verticillioides, Colletotrichum graminicola* and *Cochliobolus heterostrophus* (Gao et al., 2007), which supports the idea that *LOX3* is part of the host’s cellular components required by *U. maydis* to establish a compatible interaction. To test this hypothesis directly, a targeted mutagenesis approach was taken in the present study to knock out maize *LOX3* by employing RNA-guided Cas9 endonuclease.

Cas endonuclease technology involves a bacterial Cas9 protein guided by a clustered regularly interspaced short palindromic repeats (CRISPR)-derived, customized RNA and thus facilitates targeted genome modifications at virtually any target site of choice. Upon cleavage at the genomic target motif, the resultant DNA double-strand break is recognized and processed by the cells endogenous repair mechanisms, which, in the case of the error-prone non-homologous end-joining, entails the formation of random nucleotide insertions and/or deletions (Nishitoh et al., 2002; Puchta and Fauser, 2014). This novel principle of genetic engineering has been well established in the majority of important crop plants (Kumlehn et al., 2018; Koeppel et al., 2019). Here, Cas9 endonuclease-triggered mutagenesis of maize *LOX3* is reported. Homozygous *lox3* mutant lines are demonstrated to react stronger to pathogen-derived molecular patterns (PAMPs) by an enhanced ROS burst. In line with this, these mutants are significantly less susceptible to *U. maydis*, by which a new susceptibility factor is revealed for this biotrophic pest of maize.

## Materials and Methods

### Preparation of a *LOX3* Knockout Construct

**FIGURE 1:**
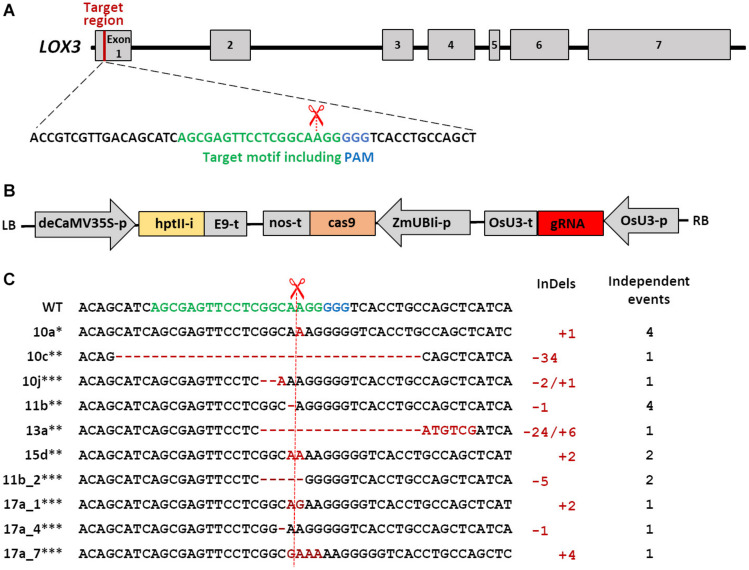
Schematic of Cas9/gRNA-mediated mutagenesis in the maize *LOX3* gene. **(A)** Schematic of *LOX3* gene structure and Cas9/gRNA target motif. Maize *LOX3* (based on B73 RefGen_v3 GRMZM2G109130) contains seven exons, represented by light gray rectangles, while introns are represented by interjacent black lines. The 19 bp specifically addressed by the gRNA are depicted in green, and the protospacer-adjacent motif (PAM) bound by Cas9 endonuclease in blue. The scissors indicate the expected cleavage site. **(B)** Schematic of the T-DNA used for plant transformation. Expression of *cas9* is driven by the maize *POLYUBIQUITIN1* promoter with first intron that resides in the 5′-UTR (UBIi). Expression of the gRNA is driven by the rice *U3* Polymerase III-processed promoter (OsU3-p). Expression of the *hygromycin phosphotransferase II* selectable marker gene including the potato *LS1* intron (hptIIi) is driven by a doubled-enhanced *CaMV35S* (deCaMV35S) promoter. E9-t, nos-t, OsU3-t: terminators; LB and RB: left and right borders of the agrobacterial T-DNA. **(C)** Site-directed mutations obtained in the *LOX3* gene. Shown is the entire variety of Cas9/gRNA-triggered *LOX3* mutation patterns detected. The plant identifiers given at the left-hand side represent also all other plants or plant families in which the same mutation pattern has independently reoccurred. ^∗^indicates mutations for which heritability was proven, ^∗∗^indicates heritable mutants whose progeny were used for infection studies, and ^∗∗∗^indicates mutations detected in T_1_ only. Deletions are highlighted with red hyphens and inserted nucleotides with red letters. The numbers of modified nucleotides and of independent events with the same mutation pattern are given at the right-hand side of the respective mutation sequences.

The *LOX3* sequence was obtained from the maize genome database^2^. The target sequence for the guide RNA (gRNA) was selected within the first exon of *LOX3* (Figure 1A) using the online platforms DESKGEN (Doench et al., 2016) and WU-CRISPR (Wong et al., 2015). Guide-RNA secondary structures were modeled using the RNAfold tool (Gruber et al., 2008). pSH121 harboring a maize codon-optimized *cas9* coding sequence under control of the maize *POLYUBIQUITIN 1* promoter and a guide-RNA scaffold preceded by the rice *U3* (RNA polymerase III-processed) promoter was used as a generic vector (Gerasimova et al., 2019). A synthetic double-stranded oligonucleotide carrying the target-specific part of the gRNA was annealed and integrated between the Os*U3* promoter and the upstream gRNA scaffold using *Bsa*I restriction and ligation. Subsequently, the fragment containing the expression cassettes of gRNA and *cas9* was transferred to the binary vector p6i-d35S-TE9 (DNA CLONING SERVICE e.K., Hamburg, Germany) using *Sfi*I restriction and ligation to generate plant transformation vector pNB104 (Figure 1B). Furthermore, the cloned vector sequences were verified by Sanger sequencing and the resultant construct introduced into the hypervirulent AGL1 strain of *Agrobacterium tumefaciens* via electroporation.

### *Agrobacterium-*Mediated Maize Transformation

Stable genetic transformation of maize was conducted using Hi-II A × B F_1_ immature embryos (Hi-II A used as female and Hi-II B used as male) as previously described (Hensel et al., 2009) with 100 mg L^–1^ hygromycin as selective agent.

### Genotyping and Mutant Verification

Maize genomic DNA was extracted from candidate transgenic and mutant plants of the T_0_ to T_3_ generations using a phenol–chloroform method as previously described (Pallotta et al., 2000). The presence of T-DNA (*cas9*/gRNA/*hpt*) in the maize genome was confirmed by PCR using specific primers (sequences in the Supplementary Table 1). Furthermore, to detect Cas9-triggered mutations in *LOX3*, the genomic region surrounding the target motif was PCR amplified using the primer pair *ZmLOX3*F1&R1 (Supplementary Table 1). PCR products were purified using the QIAquick PCR purification kit (QIAGEN, Hilden, Germany) and finally were subjected to Sanger sequencing. The sequencing files were analyzed by using the Clone Manager 9 Professional Edition (Scientific & Educational Software, Morrisville, NC, United States) and the A plasmid Editor (ApE) software. Five apparently homozygous and one heterozygous/chimeric primary mutant plants were chosen to produce and analyze further generations.

### Plant Infection

The haploid pathogenic *U. maydis* strain SG200 was used for infections. It was grown overnight in YEPS light medium (0.4% yeast extract, 0.4% peptone, and 2% sucrose) at 28°C on a rotary shaker. The culture was then diluted using fresh medium to a cell density of OD_600 nm_ of 0.2. After incubation at 28°C for about 4 to 6 h, the cells were harvested by centrifugation (10 min at 2,400 *g*) and resuspended in sterile water so that OD_600 nm_ of 1.0 was obtained. Syringe infections were made with 300 to 500 μL of the cell suspension into the interior of the leaf whorl of 7-days-old maize seedlings of wild-type and *lox3* mutants either generated by Cas9/gRNA-triggered mutagenesis or derived from transposon insertional mutagenesis (Gao et al., 2007). Three independent infections, each with about 40 plants were performed for every experiment. For quantification of disease symptoms in seedlings, a classification scheme was used according to the severity of symptoms at 8 days post-inoculation comprising seven different symptom subcategories as previously described (Kämper et al., 2006).

### RNA Isolation and Reverse Transcriptase Quantitative PCR

Leaf material was collected 4 and 8 days post-inoculation. Each biological replicate consists of leaf material pooled from 10 leaves directly frozen in liquid nitrogen and stored at −80°C. In addition, three technical replicates of each biological replicate were used for RNA isolation, cDNA preparation and reverse transcriptase quantitative PCR (RT-qPCR) analysis. Total RNA was isolated from plant tissue by using Trizol reagent (Invitrogen, CA, United States) according to the manufacturer’s instructions and stored at −80°C. The RNA quality was determined electrophoretically using a 2% non-denaturating agarose gel, and fluorometrically using a NanoDrop ND-1000 photometer. Reverse transcription was performed using the Revert Aid H Minus First Strand cDNA Synthesis Kit (Fermentas, St. Leon-Rot, Germany, K1632) with RNA (1 μg/reaction), oligo(dT)-primer (0.25 μg/reaction) and random hexamer primer (0.25 μg/reaction) according to the manufacturer’s guidelines for GC-rich templates. A total of 50 ng cDNA was used as template in a 10-μl reaction mix of the TB Green Premix Ex Taq II (TII RNase H Plus; Takara Bio Europe SAS, Saint Germain en Laye, France, RR820W) together with 0.2 μM each of forward and reverse primer. The RT-qPCR experiments were designed and conducted according to the MIQE guidelines. The reactions were performed in a LightCycler^®^ 480 (Roche Life Science, Basel, Switzerland) using the following program: 95°C, 30 s; 95°C, 5 s, 50/60°C, 30 s 72°C, 30 s (40 cycles) followed by a final melting curve with stepwise increments of 0.5°C from 65 to 95°C. Gene-specific primer sequences were retrieved from the literature (Supplementary Table 1). Maize *POLYUBIQUITIN 1* and *18S* ribosomal RNA were used as reference genes due to their reliability under various conditions according to previous findings (Shivaji et al., 2010; Manoli et al., 2012). Every primer combination was checked for its sensitivity by a primer efficacy tests using fivefold dilutions starting with 100 ng cDNA and by a melt curve to confirm the presence of no more than one transcript (Supplementary Figure 1). The geometric means of the *C*_q_ values of the two reference genes were calculated (Vandesompele et al., 2002). RT-qPCR experiments were conducted using three biological replicates, with three technical replicates per biological replicate. Raw *C*_q_ values were statistically examined using a linear mixed model described in detail by Steibel et al. (2009) and adapted in the R-Macro “qpcrmix”^3^ by calculation of log-differences of normalized gene expression data based on the 2^−ΔΔCq^ method (Livak and Schmittgen, 2001). Briefly, raw *C*_q_ data were normalized by the geometric means of two housekeeping genes (*POLYUBIQUITIN 1* and *18S*) with regard to possible random effects caused by pipetting or sampling, which resulted in Δ*C*_q_ data for each treatment of each gene as well as in *P*-values (α < 0.05) with six degrees of freedom. A linear model was applied on the Δ*C*_q_ values to quantify deviations from the two competing hypotheses that either there are no, or there are differences among the pairwise compared treatments.

### Microscopy

To evaluate fungal proliferation in infected tissue, confocal microscopy was carried out as described previously (Doehlemann et al., 2009). In brief, maize plant leaves were analyzed for 8 days after infection using the third outer leaf 1 cm below the infection site. Plant leaves were destained for at least 12 h in ethanol and incubated for 16 h at room temperature in 1M KOH. Further, the samples were gently washed 3 times with 50 mM Tris (pH 7.5). Fungal hyphae were stained with 10 mg/mL wheat germ agglutinin (WGA)-Alexa Fluor 488 conjugate (Molecular Probes, OR, United States), while plant cell walls were visualized using 1 mg/mL propidium iodide (Sigma-Aldrich, MO, United States)/0.02% Tween 20 for 30 min, followed by washing with 50 mM Tris at pH 7.5. The resulting samples were carefully analyzed using a Zeiss LSM780 confocal laser microscope (Carl Zeiss, Jena, Germany). The plant cell wall was visualized by a 561 nm laser with an emission spectrum of 584–651 nm. Fungal hyphae were visualized by WGA-Alexa Fluor signal using a 488 nm laser and an emission spectrum of 493–541 nm. Fluorescence induction was obtained by means of sequential scanning. Pictures represent maximal *z*-stack projections. Captured images were further processed using the ImageJ software.

### Quantification of *U. maydis* Biomass

Biomass quantification was carried out as described (Brefort et al., 2014) to determine the differences between wild-type and maize *lox3* mutants; 7-days-old maize seedlings were infected with SG200. Six days post-inoculation, a 2-cm section from the tip of the 3^*rd*^ leaf was used for analysis. Similarly, the same region of the 4^th^ leaf was used 12 days post-inoculation. Ten leaf segments were pooled per each of the indicated points in time and the experiment was performed using four biological replicates. For genomic DNA extraction, leaf material was frozen in liquid nitrogen, ground to powder, and extracted using a phenol-based protocol (Pallotta et al., 2000). The quantitative PCR (qPCR) analysis was performed using a LightCycler^®^ 480 (Roche Life Science, Basel, Switzerland) in combination with the SYBR Premix Ex Taq (TII RNase H Plus) (Takara Bio Europe SAS, Saint Germain en Laye, France). *U. maydis* biomass was quantified using primers specific for the fungal *Peptidyl-prolyl isomerase* (*Ppi*) gene. The maize *GLYCERALDEHYDE 3-PHOSPHATE DEHYDROGENASE* (*GAPDH*) served as reference gene for normalization (Supplementary Table 1). Relative amounts of fungal DNA represented by amplified *Ppi* were then calculated relative to the amount of maize-derived GAPDH DNA using the cycle threshold (Ct) 2^−2^*^C^*^t^ method.

### Quantification of PAMP-Triggered ROS Accumulation

ROS accumulation was measured in maize plants using a luminol-based bioassay as described (Hilbert et al., 2013; Hückelhoven and Seidl, 2016; Navarrete et al., 2019; Samira et al., 2019). This assay is relying on the detection of the luminescence released by excited luminol molecules produced after horseradish peroxidase (HRP)-catalyzed oxidation of luminol molecules in the presence of plant-derived ROS. The emitted light directly correlates to the amount of H_2_O_2_ produced upon PAMP-treatment of the plant. Maize plants were grown in a climate chamber at 16/8 h light/dark cycles at 25/18°C in peat moss-based substrate. Six days after germination, plants were infected with the solo-pathogenic *U. maydis* strain SG200. Four days post-inoculation, eight leaf disks were collected from the midrib of the third leaf using a biopsy punch, and incubated in a black 96-well polystyrene plate containing 100 μL of deionized water. The plates were then covered with aluminum foil and incubated overnight at room temperature. Water was removed and flagellin (flg22) solution was added which comprised Horseradish peroxidase (HRP 10 μg/mL, Sigma-Aldrich cat# P8375), L-012 (34 μg/mL Fujifilm WAKO cat# 120-04891) and flg22 (100 nM) in H_2_O. The production of reactive oxygen species was monitored by luminescence over 30 to 40 min in a microplate reader (Spark, Tecan). At least three plants per mutant were used in each experiment. All experiments were performed at least 4 times.

### Institutional Permits to Work With Genetically Engineered Materials

All of the experiments associated with genetic engineering were conducted in laboratories or under glasshouse conditions certified as biosafety level 1.

### Statistical Analyses

RT-qPCR/qPCR data were calculated using a previously published R-macro (Steibel et al., 2009). For the evaluation of disease scoring, an R−script was used to process the data. Class counts were summarized for each genotype across the three biological replicates. For each pairwise comparison of genotypes, Fisher’s exact test was applied. This test calculates single *P*-values of each treatment across all symptom subcategories. The *P*-values were multiple testing-corrected by the Benjamini–Hochberg algorithm (Benjamini and Hochberg, 1995). For the figures, the counts for each treatment were converted into relative values. For the case of Supplementary Figure 3, *P*-values were calculated by the parameter-free Wilcoxon rank-sum test. The fungal biomass data were processed by the Student’s *t*-test.

## Results

### Molecular Characterization of Maize *LOX3* Mutations and Their Generative Transmission

The target motif addressed by RNA-guided Cas9 endonuclease was selected within the first exon of *LOX3*. In addition, a detailed off-target analysis was performed using the DESKGEN platform (Doench et al., 2016) (Supplementary Figure 2), which revealed potential off-targets with at least three base pair mismatches. However, off-target cleavage is very unlikely in motifs with three or more base pair mismatches. From a total of 140 Agrobacterium-infected Hi-II A x B immature embryos, 88 putative primary transgenic plants were generated, all of which were proven by transgene-specific PCR analyses for *CRISPR-associated* 9 (*cas9*) endonuclease, gRNA and *hygromycin phosphotransferase* (*hpt*) to carry the T-DNA derived from the transformation vector pNB104 (Figure 1B). From all but three of these primary transgenic (T_0_) plants, amplicon sequences were obtained from the target region. All these 85 plants carried mutations in the target motif. According to the amplicon sequences, 82 of these mutants were putative homozygous (containing no more than one mutant and no wild-type allele), whereas the remaining three mutant plants were heterozygous and/or chimeric with the wild-type allele being present (i.e., at least one mutant and the wild-type allele). However, the presence of additional alleles cannot be ruled out, because a given leaf sample used for genomic DNA extraction and amplicon sequencing does not necessarily represent the entire plant. The vast majority of mutations were one- and two-nucleotide insertions (46 and 19%, respectively) and one-nucleotide deletions (13%). In addition, deletions of as many as 34 nucleotides and combined insertions/deletions were among the mutant alleles. The variety of mutation patterns detected is shown in Figure 1C.

To provide evidence for generative transmission of mutant alleles, progenies derived from selected T_0_ plants were also subjected to target motif analysis. As expected for chimeric mother plants, some mutations were not inherited by the T_1_ plants (#17a, Figure 1C). Most likely owing to ongoing Cas9 activity and still present wild-type alleles after analysis of T_0_ plants, also some new mutations occurred in their progeny (#11b-2). The presence/absence of the T-DNA in progeny of six selected primary mutants was determined by PCR using 10 T_1_ plants each, which revealed T-DNA-free segregating siblings being present in five of these families. If not already given in T_0_, homozygosity for the mutations was achieved by self-pollination. All *lox3* mutant plants did not show any visual phenotypic abnormalities of their above-ground parts under the glasshouse conditions used in this study.

### *lox3* Mutants Show Moderate Resistance to *U. maydis*

**FIGURE 2:**
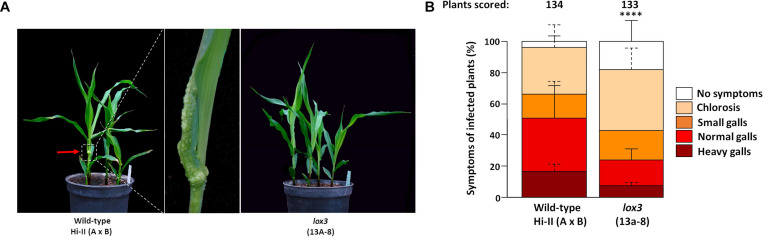
Disease rating of Cas9/gRNA-triggered *LOX3* mutant and WT plants infected with the solo-pathogenic *U. maydis* strain SG200 at 8 days post-inoculation (dpi). **(A)** Phenotype of *lox3* mutants and wild-type (WT) plants in response to *U. maydis* infection at 8 dpi. Middle: Heavy gall formation, as indicated by a red arrow, was observed significantly more frequently on WT than on infected *lox3* mutant plants. **(B)** Corn smut disease rating on WT versus *lox3* mutant maize as scored 8 dpi. *P*-values were calculated by Fisher’s exact test. Multiple testing correction was done by the Benjamini-Hochberg algorithm. Error bars indicate the standard errors of the means of relative counts from three replicates. Every second error bar is dotted to facilitate the discrimination of overlapping ones. Asterisks indicate the significant difference (*P* < 0.0001) between mutant and WT across all symptom subcategories.

An experiment was carried out to determine the response of maize *lox3* mutants to *U. maydis* infection. T_2_ siblings derived from a homozygous mutant T_1_ line (#13a-8 with a +24/-6 nucleotides indel) were infected with freshly grown *U. maydis*, with azygous wild-type plants (derived from the same tissue culture procedure) being used as control. Following injection using the fungal cell suspension, disease symptoms ranging from chlorosis via light swelling up to heavy gall formation on the aerial parts of the maize plant appeared within one week. Disease symptoms were scored at day 8 post-inoculation. The size and shape of the galls varied remarkably between the wild-type and mutant plants (Figure 2A). Mutant plants were less susceptible to *U. maydis* infection, as around 20% of inoculated plants were asymptomatic, while the majority of symptomatic plants exhibited weak (38%) or moderate symptoms (17%) and only 8% showed heavy symptoms (Figure 2B). In contrast, wild-type maize exhibited significantly stronger infection symptoms (Figure 2B). A further analysis was carried out with T_2_ siblings of the three independent mutant lines #15d-8, #11b-9, and #10c-1 (with +2, −1, and −34 indels, respectively) for their susceptibility toward *U. maydis* infection. Selected mutant plants also showed decreased disease severity to *U. maydis* infections in comparison to wild-type (Supplementary Figure 3). Furthermore, *lox3* mutants with B73 background generated by transposon insertional mutagenesis (Supplementary Figure 4A) also exhibited significantly decreased disease severity to *U. maydis* infections (Supplementary Figure 4B), which provides convergent evidence that loss-of-function of *LOX3* renders maize moderately resistant to *U. maydis*.

### *lox3* Mutants Exhibit Reduced Fungal Biomass

**FIGURE 3:**
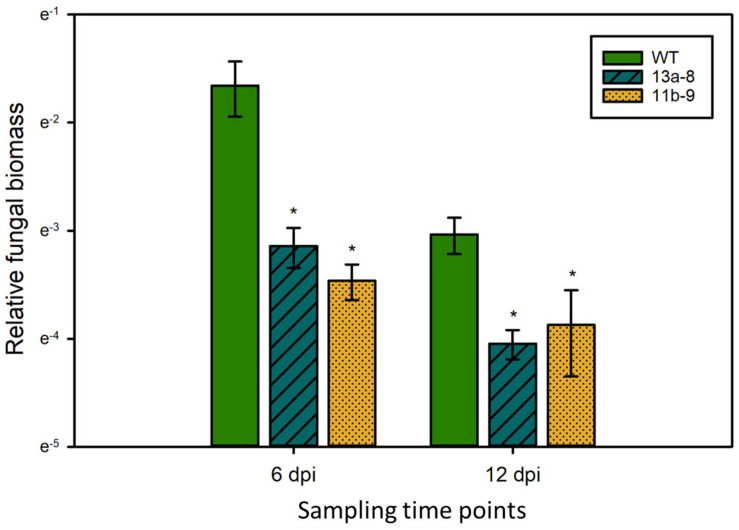
Quantification of *U. maydis* biomass. Genomic DNA was extracted from maize leaves 6 and 12 dpi with *Ustilago maydis* strain SG200 and used for qPCR. Relative fungal biomass was calculated by the comparison between the *U. maydis Peptidylprolyl isomerase* (*Ppi*) and the *Z. mays GLYCERALDEHYDE 3-PHOSPHATE DEHYDROGENASE* (*GAPDH*) genes. Data represent three biological replicates, with three technical replicates for each biological replicate. Error bars indicate standard deviations. Significant differences (Student’s *t*-test, *P* < 0.05) between mutant and wild-type are indicated by asterisks.

To test if the observed differences in symptom formation upon *U. maydis* infection of wild-type and *lox3* mutant plants were indeed due to lower colonization by the fungus, a fungal biomass quantification was performed by qPCR assessing the abundance of fungal genomic DNA in a defined amount of infected plant tissue. The fungal biomass was significantly less in the *lox3* mutants at 6 and 12 days post-inoculation in comparison to infected wild-type maize (Figure 3).

### No Obvious Differential Inter- and Intracellular Fungal Growth Features of *U. maydis* in Wild-Type and *lox3* Mutant Plants

**FIGURE 4:**
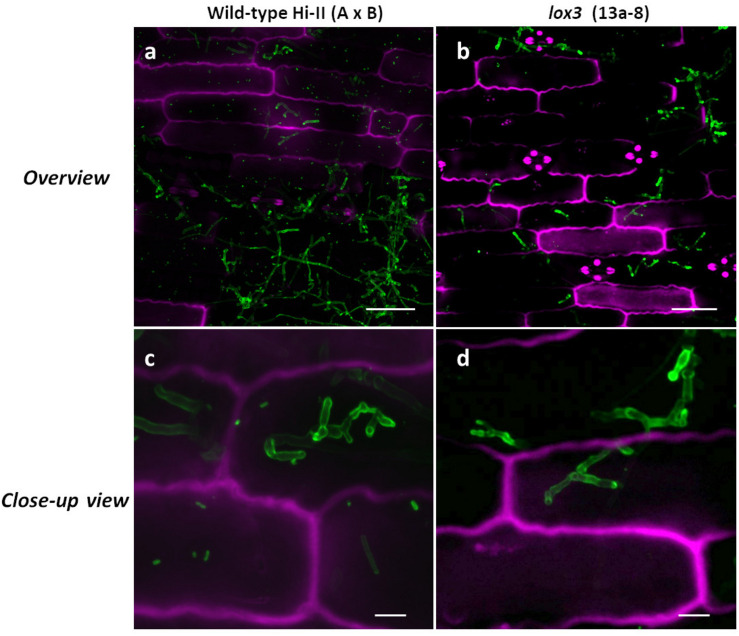
Confocal microscopic examination of *U. maydis-*infected tissue in wild-type and *lox3* mutant (line #13a-8) maize 8 dpi. *U. maydis* invasive inter- and intracellular growth and formation of branching hyphae. Infected plant tissue was stained with propidium iodide (magenta) and fungal hyphae with lectin-binding WGA-AF488 (green). Scale bars in pictures **(a)** and **(b)** = 50 μm; scale bars in **(c)** and **(d)** = 10 μm

Confocal microscopy was used to visualize inter- and intracellularly growing fungal hyphae comparing wild-type and *lox*3 mutant (line #13a-8) plants infected with *U. maydis*. Whereas disease symptom scoring showed quantitative differences, microscopy did not reveal any obvious differences in the hyphal structure or the infected tissues when comparing wild-type with *lox3* mutant plants (Figure 4).

### Infection-Dependent Regulation of Maize *9-LOX* and Associated Gene Expression

**FIGURE 5:**
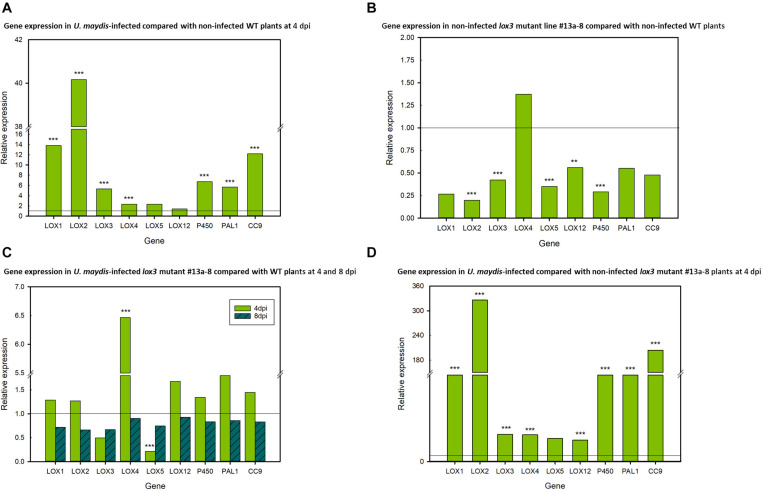
Differential relative expression of selected *9-LOX* and associated genes. **(A)** Relative gene expression in *U. maydis*-infected and non-infected wild-type (WT) maize plants at 4 dpi, with the WT expression being set to 1. **(B)** Relative gene expression in non-infected *lox3* mutant #13a-8 and non-infected WT maize at 4 days after mock-inoculation, with expression of the non-infected WT being set to 1. **(C)** Relative gene expression in *U. maydis*-infected *lox3* mutant #13a-8 and WT maize plants at 4 and 8 dpi, with the WT expression being set to 1. **(D)** Relative gene expression in *U. maydis*-infected and non-infected *lox3* mutant #13a-8 maize plants at 4 dpi, with the expression in non-infected plants being set to 1. The experiments were conducted using three biological replicates, with three technical replicates for each biological replicate. Asterisks indicate significant differences between treatments at *P* < 0.01 (**) and 0.001 (***). *P*-values were calculated by a previously published R-Macro (Steibel et al., 2009).

To investigate the transcriptional regulation of selected *9-LOX* (*LOX1*, *LOX2*, *LOX3*, *LOX4*, *LOX5*, *LOX12*), associated (*P450*, *CORN CYSTATIN-9* (*CC9)* and *PHENYLALANINE AMMONIA LYASE* (*PAL*) genes in maize in response to *U. maydis* infection, their transcript amounts were analyzed by RT-qPCR. Transcripts of the selected genes were measured in infected vs. non-infected leaf tissue (using *lox3* mutant line #13a-8, Hi-II A x B) at two points in time, namely at days 4 and 8 post-inoculation. The results illustrate that the expression of the 9-*LOX*s *LOX1*, *LOX2*, *LOX3*, *LOX4* and of the associated genes *CC9*, *PAL*, *P450* was significantly upregulated in infected wild-type plants in comparison to their non-infected (mock-treated) wild-type counterparts. Showing the same trend, *LOX5* and *LOX12* were also upregulated, albeit not with statistical significance (Figure 5A). Remarkably, *LOX2*, *LOX3*, *LOX5, LOX12* and *P450* transcripts were significantly down-regulated in the mock-treated mutants as compared to mock-treated wild-type plants. Furthermore, reduced transcripts were also observed in the *CC9*, *PAL* and *LOX1* genes, which was, however, not statistically significant (Figure 5B). We continued further by measuring the transcripts in infected mutant plants. At 4 days post-inoculation, only *LOX4* was significantly upregulated and *LOX5* significantly down-regulated in the *lox3* mutant plants. Although not significant, *LOX3* transcripts showed lower levels in comparison to infected wild-type plants. Interestingly, at 8 days post-inoculation, transcripts of all genes showed a tendency of down-regulation, but non-significant (Figure 5C). Very similar to wild-type plants (Figure 5A), also *lox3* mutants show largely consistent upregulation of these genes upon infection by *U. maydis* (Figure 5D).

### *lox3* Mutant Maize Responds With Increased ROS Accumulation to PAMPs

**FIGURE 6:**
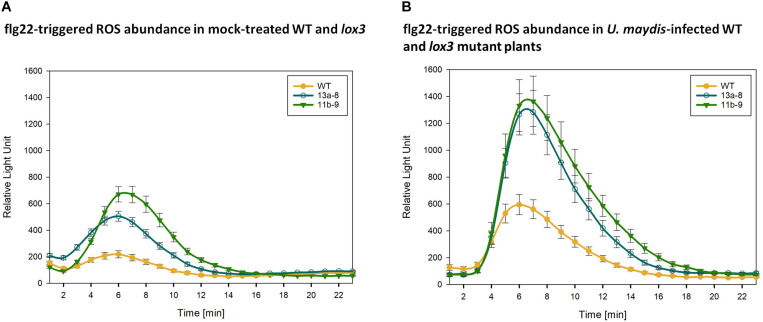
PAMP-triggered ROS accumulation. Quantification of flg22-triggered H_2_O_2_ abundance in mock-treated **(A)** and *U. maydis* infected **(B)** Cas9/gRNA-triggered *lox3* mutants and wild-type (WT) plants. Curves represent H_2_O_2_ levels monitored by a luminol-based peroxidase assay where luminescence directly correlates to H_2_O_2_ levels of the plant over time. As compared to WT, the mutant lines #13a-8 and #11b-9 show higher ROS accumulation. Shown are the average values ± standard error of the means of four independent experiments.

To find an explanation for the moderate resistance of *lox3* mutant maize toward *U. maydis*, various early host defense responses were tested upon infection with *U. maydis*. One of the first signaling and defense responses that plants activate upon recognition of invading microbes is the accumulation of ROS in the apoplastic space, a process that is usually suppressed by effectors from virulent pathogens (Jones and Dangl, 2006; Dodds and Rathjen, 2010). We assessed the ROS abundance in wild-type and *lox3* mutants in response to the standard PAMP flagellin and *U. maydis* infection. To this end, leaf disks of plants were treated with the PAMP flg22 and ROS production was monitored over 30 to 40 minutes using a luminol-based assay. A clear difference was observed in ROS production; *lox3* mutants exhibited an enhanced PAMP-triggered ROS burst in comparison to the wild-type maize plants. This was observed upon flagellin treatment alone (Figure 6A) and, even more pronounced, in response to additional infection by *U. maydis* (Figure 6B). The enhanced ROS-accumulation in *lox3* mutant maize and the corresponding PTI responses might be the basis of the reduced colonization success of *U. maydis*.

## Discussion

Cas endonuclease technology has evolved as a powerful means to improve crop plants through site-directed genome modification. For the improvement of plant disease resistance, this approach has been employed to target susceptibility factors. For instance, targeted mutagenesis of tomato *DMR6* entailed resistance to a variety of pathogenic *Pseudomonas, Phytophthora* and *Xanthomonas* species (De Toledo Thomazella et al., 2016), and of wheat *EDR1* to powdery mildew (Zhang Y. et al., 2017). In the present investigation, the putative susceptibility gene *LOX3* of maize was knocked out by Cas9-triggered mutagenesis. Out of the 88 primary Cas9/gRNA-transgenic maize plants generated, mutated target motifs were found in all but three. In the latter, however, PCR failed to produce amplicons from the target region, suggesting that modifications might have been so large in these plants, that at least one of the primer sites was affected. The achieved mutagenesis efficiency rounded off to 97% is on a par with the best results reported thus far in maize (Shi et al., 2017). The predominant occurrence of small insertions and deletions amongst the mutations obtained is in accordance with previous work on crop species of the *Poaceae* family as well (Shi et al., 2017; Gerasimova et al., 2020). Based on a comparison of a choice of T_0_ plants with their (T_1_) progenies, it was demonstrated that the vast majority of Cas9/gRNA-triggered mutations were of heritable nature. However, the variety of mutation patterns found within T_1_ siblings indicates that some mutations and even residual wild-type sectors have remained undetected by analyses of leaf samples, which can be explained by mosaicism of the respective T_0_ plants. Moreover, the presence of wild-type alleles in heterozygous or chimeric mutant T_0_ plants expressing the *cas9* and gRNA transgenes has likely resulted in additional mutations triggered only after the samples were taken from the T_0_ plantlets. In 5 out of 6 T_1_ families analyzed, non-transgenic siblings bearing a *lox3* mutant allele in fixed condition were identified. All these findings were in accord to what has been observed in previous investigations in maize and other plants (Schedel et al., 2017; Li et al., 2019; Budhagatapalli et al., 2020).

The generated Cas9/gRNA-triggered mutants did not exhibit any morphological differences in their above-ground parts as compared to wild-type plants. In this context, some background variation in plant size among segregating siblings owing to the hybrid nature of the T_0_ plant was observed. By contrast, the previously described transposon insertion-based *lox3* mutants showed increased attractiveness to root-knot nematodes (Gao et al., 2008) indicating a potential for unwanted pleiotropic effects. Off-target mutations induced by the employed Cas9/gRNA complexes may also lead to unintended effects. However, by selecting target motifs that do not have any identical copies in the maize genome, the occurrence of unintended mutations was largely ruled out in the present investigation. Moreover, the increased resistance to *U. maydis* of the Cas9/gRNA-triggered mutants was independently confirmed by the analysis of *lox3* mutants generated by transposon insertion.

Mutant line #13a carries an insertion of 6 bp along with a deletion of 24 bp in exon 1 of the target gene, which constitutes a loss of 6 amino acids in the gene product, with the translational reading frame being retained. This mutation could have certainly lead to a still functional allele. However, the disease scoring data presented in Figure 2 provide compelling evidence for the altered behavior of this mutant, which suggests that the *LOX3* gene function is at least strongly reduced, if not entirely abolished.

*LOX3* belongs to the 9-lipoxygenases (Wilson et al., 2001), while *LOX*s constitute a large gene family of non-heme iron-containing fatty acid dioxygenases which are ubiquitous in plants and animals (Feussner and Wasternack, 2002). In plants, LOXs catalyze the incorporation of molecular oxygen into free fatty acids, primarily linoleic (C18:2) and linolenic (C18:3) acids, either at position 9 or 13 of their carbon chains and, therefore, are referred to as 9-LOXs or 13-LOXs, respectively. 9-LOXs produce various oxylipins such as 9-hydroxyoctadecadienoic acid (9-HODE), 9-keto-octadecadienoic acid (9-KODE), and 9-keto-octadecatrienoic acid (9-KOTE) (Gao et al., 2008). Certain fungi exploit specific host 9-LOXs and their derivatives to facilitate pathogenesis (Brodhagen and Keller, 2006; Sagaram et al., 2006; Tsitsigiannis and Keller, 2007). For instance, expression of maize *LOX3* was induced by *Fusarium verticillioides* and *Aspergillus flavus* in lines that accumulate particularly high levels of mycotoxins (Wilson et al., 2001). Consistently, the inactivation of maize *LOX3* by insertion of a transposable element led to a reduction of the disease severity upon *F. verticillioides*, *Colletotrichum graminicola*, and *Cochliobolus heterostrophus* infections (Gao et al., 2007). The specific chemical functions of the *9-LOX* genes are largely unknown. On the other hand, literature indicates that 9-oxylipins likely regulate JA production in maize (Borrego and Kolomiets, 2016). This is corroborated by the observation that some 9-LOXs possess dual substrate specificity by catalyzing 9- as well as 13-hydroperoxides (HPODs). For instance, Kim et al. (2003) demonstrated that maize *LOX1* produces 13-hydroperoxylinolenic acid and 9-hydroperoxylinolenic acid in a 6-to-4 ratio. 13-hydroperoxylinolenic acid is an intermediate substrate in the JA biosynthesis pathway. For maize, this suggests a role of *LOX1* in JA regulation. As another predominant 9-LOX, maize LOX12 appears to act as a positive regulator of JA production (Christensen et al., 2014). The most compelling indications for a role of maize LOX3 in JA biosynthesis come from Gao et al. (2008) who have demonstrated that maize *lox3* mutant plants show a tendency to have lower JA levels in the leaves and a corresponding increase of salicylic acid (SA). This correlation could help explain why *U. maydis* is hampered in establishing biotrophy in maize *lox3* mutants, since elevated SA levels have been shown previously to inhibit fungal colonization (Djamei et al., 2011).

Four days after infection, transcripts of the maize *9-LOX* genes *LOX1, LOX2, LOX3, LOX4, LOX5* as well as *P450* were upregulated upon *U. maydis* infection and this data aligns with what has been previously observed (Doehlemann et al., 2008), suggesting that *U. maydis* manipulates the expression of these *9-LOX* genes to facilitate colonization. Similarly, expression of maize *LOX3* was demonstrated to facilitate infection by *F. verticillioides* (Wilson et al., 2001; Gao et al., 2007). Furthermore, upregulated expression of *LOX1* and *LOX5* promotes *Fusarium graminearum* infection (Nalam et al., 2015). In the present investigation, transcripts of maize *LOX1, LOX2, LOX3, LOX4, LOX5*, and *LOX12* were down-regulated at day 8 post-inoculation in *U. maydis*-infected mutants as compared with infected wild-type counterparts. Whether the lack of 9-LOX products of the different *LOX* genes including *LOX3* is causing reduced susceptibility or the reduced *9-LOX* transcripts are a consequence of reduced colonization due to the lack of a specific *LOX3* product remains an open question. Maize *LOX12* (which is classified as a *9-LOX*) and *LOX5* were not significantly upregulated in infected versus mock-treated wild-type plants 4 days post-inoculation. In the *F. verticillioides*-maize interaction, *LOX12* was previously shown to be important for JA-mediated responses (Christensen et al., 2014). By contrast, we observed differential regulation of *LOX4* and *LOX5* transcripts in infected *lox3* mutants versus infected wild-type plants 4 days post-inoculation. However, 8 days post-inoculation, all transcripts tested showed a trend of lower levels in the *lox3* mutant plants. This could itself be a consequence of the overall reduced fungal proliferation owing to the loss-of-function of the *LOX3* susceptibility gene.

*CC9* is a known compatibility factor for the biotrophic interaction of maize with *U. maydis*, as *CC9*-silenced maize plants featured penetration resistance (van der Linde et al., 2012). Consequently, *CC9* can be used as a marker gene for JA-related responses (Pinter et al., 2019). However, neither 4 nor 8 days post-inoculation, the comparison between wild-type and *lox3* mutant plants infected with *U. maydis* showed significant differences in *CC9* transcript levels. This suggests either that JA signaling induction upon *U. maydis* is not hampered or that *U. maydis* induces host *CC9* transcripts in JA-independent manner. Doehlemann et al. (2008) demonstrated that *PAL* transcript levels were strongly increased in gall tissue 8 days post-inoculation. Similarly, a significant transcript upregulation was observed in the wild-type plants 4 days post-infection in the present study. A tendency of reduced *PAL* transcription was observed in non-infected mutant plants in comparison to the wild-type 4 days after mock-inoculation. A similar trend was observed in infected *lox3* mutant compared with wild-type plants at 8 days post-inoculation. Under consideration that *PAL* was reported as being activated by the JA/ET signaling pathway (Diallinas and Kanellis, 1994; Kato et al., 2000; Shoresh et al., 2005), the reduced transcript abundance at 8 days post-inoculation suggests that JA/ET signaling is compromised in the *lox3* mutants.

To understand the biochemical mechanisms behind increased resistance observed in *lox3* mutants, ROS production was measured. Typically, ROS act as cellular signaling molecules to trigger plant immune responses, such as PTI and effector-triggered immunity (Jwa and Hwang, 2017). To stop the fungal spread, plants accumulate ROS which promote some defense responses that can culminate in localized cell death. Plants use this defense strategy against biotrophs and hemi-biotrophs (Constantino et al., 2013; McCormick, 2017). In agreement with this, *lox3* mutant plants exhibited an enhanced ROS burst, suggesting that PAMP-triggered immunity is activated against *U. maydis.* Constantino et al. (2013) reported that *lox3* maize mutants (generated via transposon insertional mutagenesis) accumulated higher levels of ROS in comparison to the wild-type at 24 h post-inoculation with *C. graminicola.* They proposed that this increase likely limits the duration of the biotrophic stage of the fungal life cycle in the course of the disease, suggesting a role of lipoxygenases in the regulation of ROS. There is compelling evidence that *U. maydis* inhibits the plant oxidative burst to establish the biotrophic interaction (Molina and Kahmann, 2007; Hemetsberger et al., 2012; Navarrete et al., 2019). For instance, Molina and Kahmann (2007) speculated that virulence of *U. maydis* depends on its ability to detoxify ROS. Furthermore, Hemetsberger et al. (2012) showed that the *U. maydis* effector Protein essential during penetration 1 (Pep1) suppresses plant immunity by inhibition of host peroxidase activity. Additionally, *U. maydis* employs a whole cluster of effectors, the Pleiades which all show ROS burst suppressive activity *in planta*. The above-discussed results prompt us to postulate, that higher accumulation of ROS in *lox3* mutants likely limits the infection and proliferation success of *U. maydis*, which might be the reason for the moderate resistance in *lox3* mutant maize.

## Conclusion

In the present study, *lox3* mutants were engineered by Cas9 endonuclease technology. These mutants featured moderate resistance to the corn smut fungus *U. maydis*. This observation was then corroborated by transposon insertional *lox3* mutants showing the same response as their Cas9-triggered counterparts. *lox3* mutant plants did not show any visual phenotypic abnormalities with regard to their above-ground parts under the glasshouse conditions used in this study. Consequently, the loss-of-function of this susceptibility factor holds some promise for the opportunity to breed maize cultivars with enhanced resistance to fungal pathogens. However, more detailed investigations are needed yet to analyze *lox3* mutant performance against pathogenic insects and other fungi and to rule out any substantial trade-off effects that might be associated with these mutants.

## Data Availability Statement

All datasets presented in this study are included in the article/Supplementary Material.

## Author Contributions

JK conceived the study. AD supervised the plant–fungus interaction part. KP, PR, RB, and IS performed the experiments. KP, NB, and MB analyzed the data. SH designed and generated vector pSH121. KP, AD, and JK wrote the manuscript. All authors amended the manuscript.

## Conflict of Interest

The authors declare that the research was conducted in the absence of any commercial or financial relationships that could be construed as a potential conflict of interest.

## Footnotes

1 http://faostat.fao.org

2 https://www.maizegdb.org/

3 https://github.com/daniel-gerhard/qpcrmix

## Supplementary Material

Supplementary Figure 1 Efficiency and melt curve analysis of the primers used for RT-qPCR.

Supplementary Figure 2 Target motif evaluation using the DESKGEN online platform. On-target and off-target scores were labeled with red boxes. The summary of off-target analysis is indicated by a green box.

Supplementary Figure 3 Corn smut disease rating on WT versus Cas9/gRNA-triggered *lox3* mutants in maize as scored at 8 dpi. T_2_ plants were used for the infection assays. *P*-values were calculated by the parameter-free Wilcoxon rank-sum test. Multiple testing correction was done by the Benjamini-Hochberg algorithm. Asterisks indicate significant differences as compared with the WT at the level of *P* < 0.05.

Supplementary Figure 4 **(A)** Schematic of Mu transposon insertion site in *LOX3.* **(B)** Corn smut disease rating on WT versus the *lox3* mutant plants (generated via transposon insertion) as scored 8 dpi. Standard errors of the means of relative counts from three replicates are displayed. Every second error bar is dotted to facilitate the discrimination of overlapping bars. *P*-values were calculated by Fisher’s exact test. Multiple testing correction was done by the Benjamini-Hochberg algorithm. Asterisks indicate significant differences as compared with WT at the level of *P* < 0.0001.

Supplementary Table 1 PCR, RT-qPCR, and qPCR primers used in this study.
