# Supplementary figures and images for: Engineering Smut Resistance in Maize by Site-Directed Mutagenesis of LIPOXYGENASE 3
Source: Front Plant Sci. 2020 Oct 21;11:543895. doi: 10.3389/fpls.2020.543895 (PMC7609844; doi:10.3389/fpls.2020.543895)

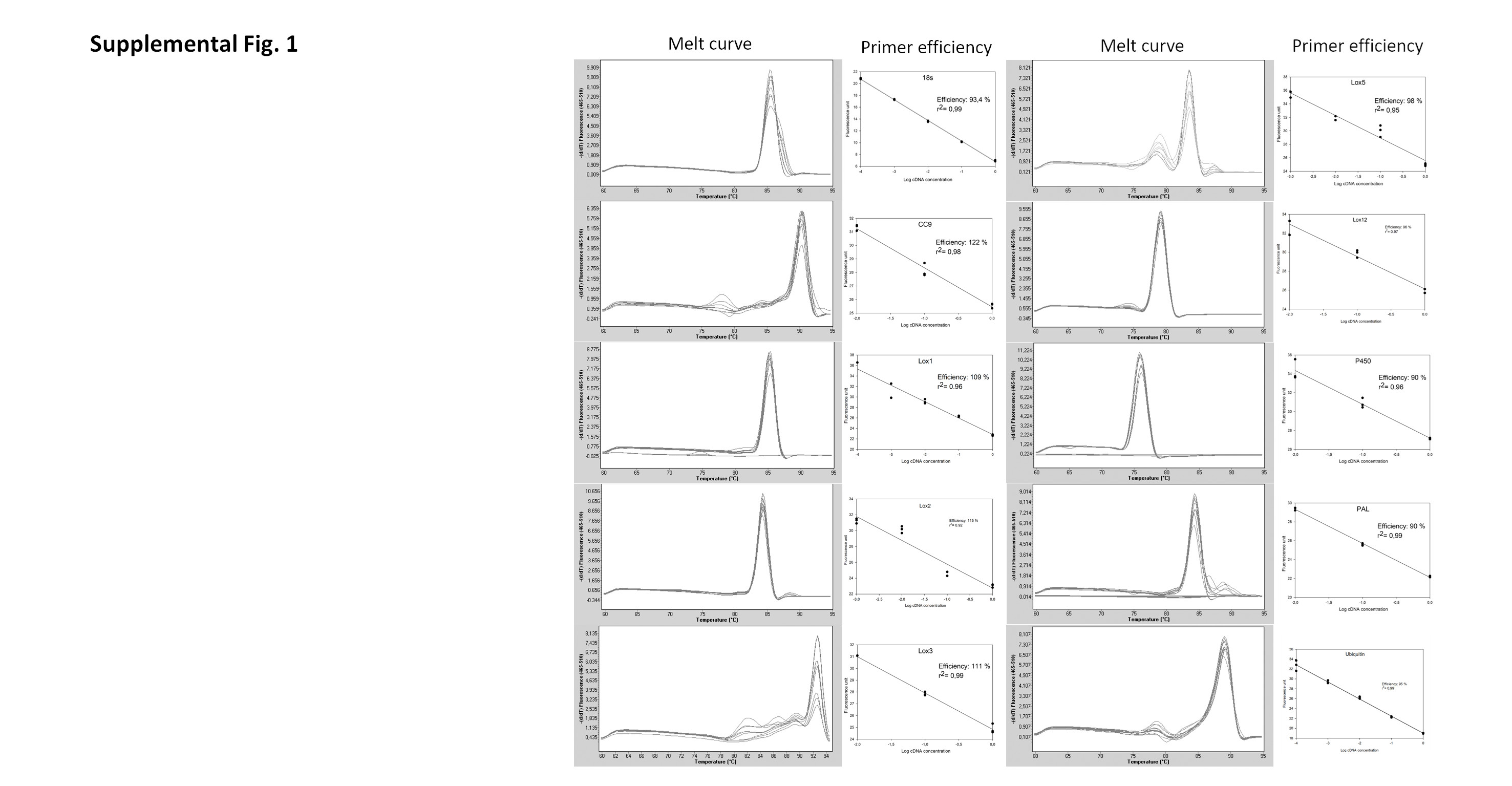

Supplement: Supplementary Figure 1 — Efficiency and melt curve analysis of the primers used for RT-qPCR. [file Image_1.JPEG]

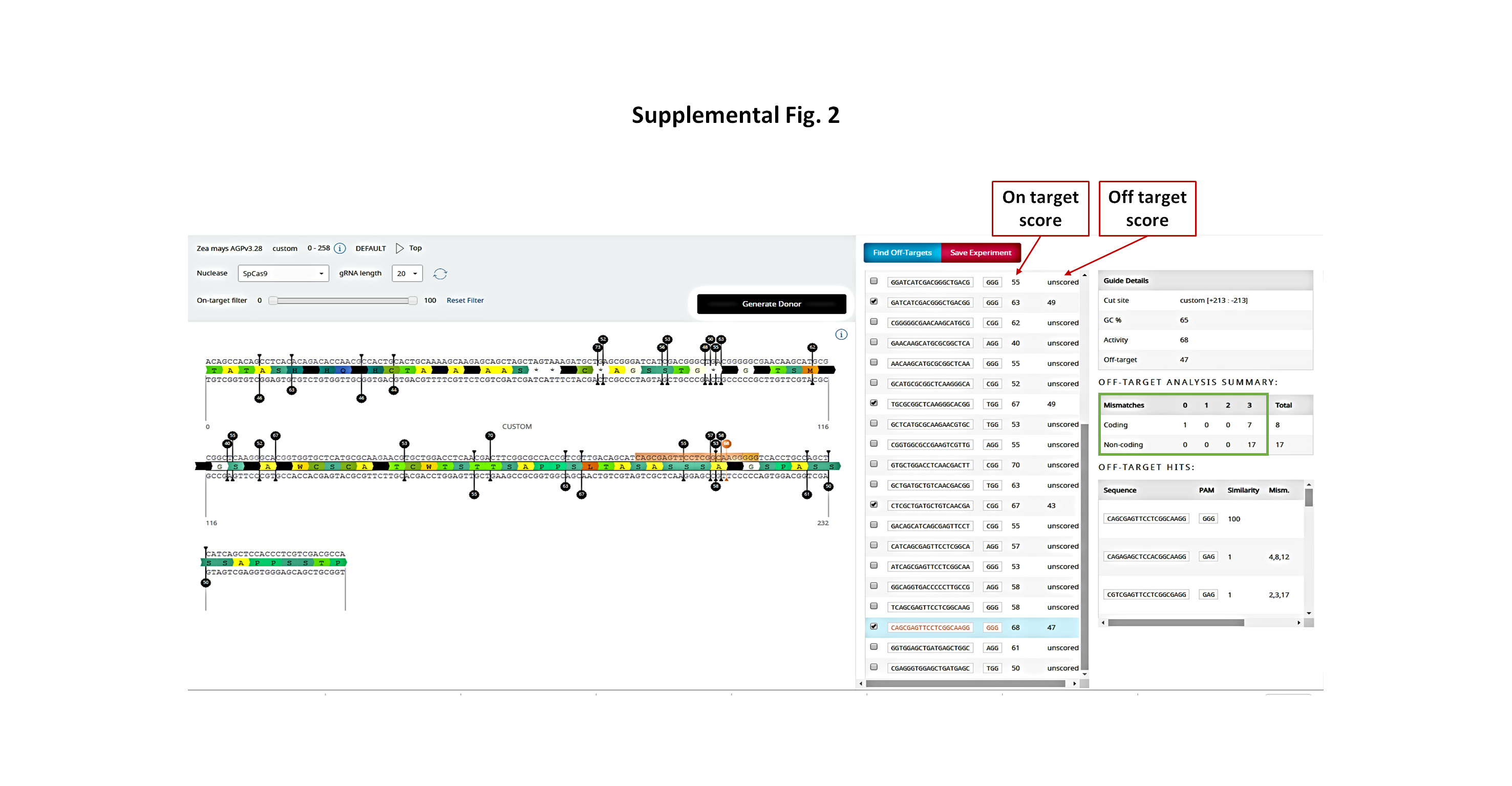

Supplement: Supplementary Figure 2 — Target motif evaluation using the DESKGEN online platform. On-target and off-target scores were labeled with red boxes. The summary of off-target analysis is indicated by a green box. [file Image_2.JPEG]

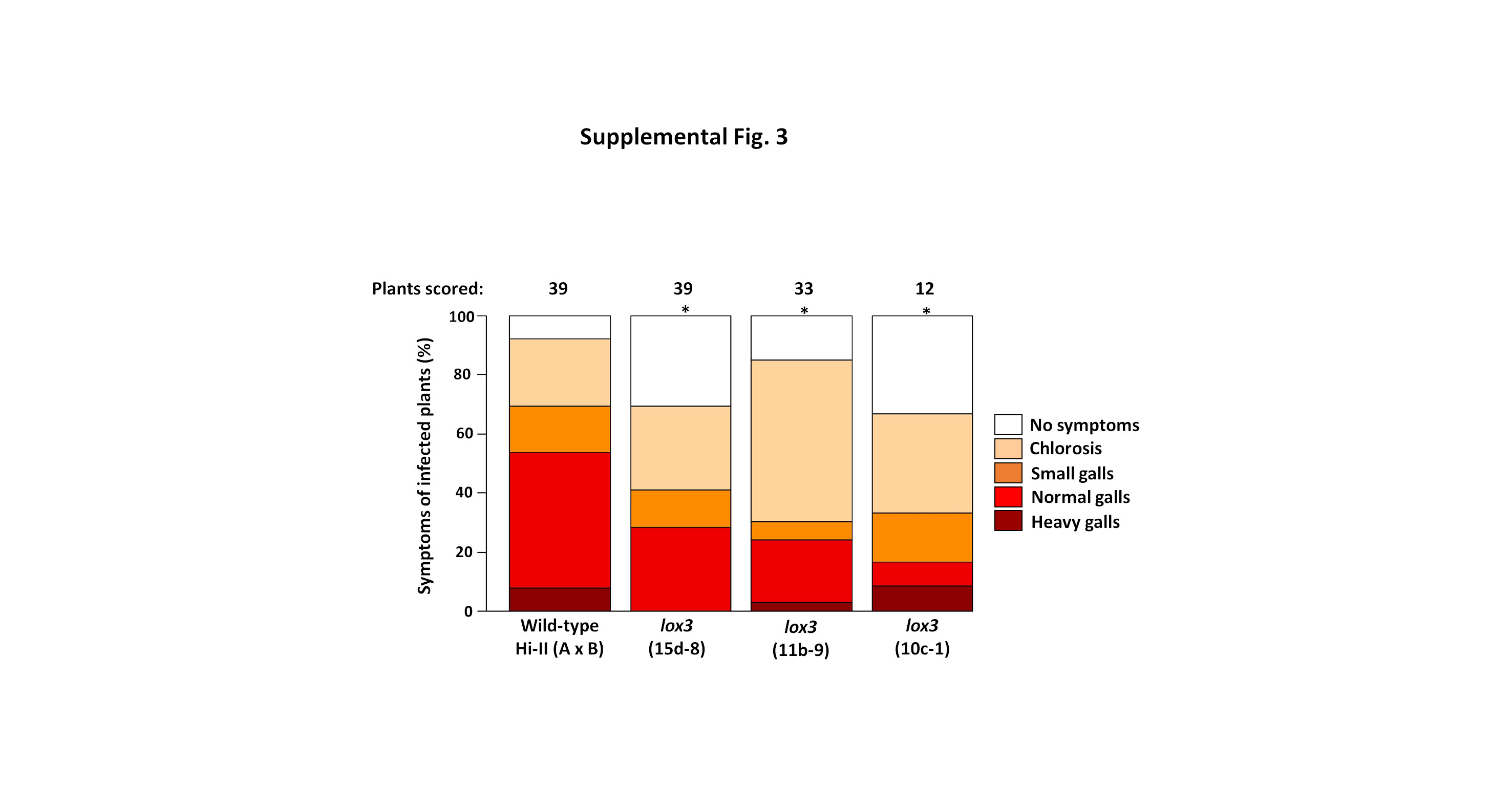

Supplement: Supplementary Figure 3 — Corn smut disease rating on WT versus Cas9/gRNA-triggered lox3 mutants in maize as scored at 8 dpi. T2 plants were used for the infection assays. P-values were calculated by the parameter-free Wilcoxon rank-sum test. Multiple testing correction was done by the Benjamini-Hochberg algorithm. Asterisks indicate significant differences as compared with the WT at the level of P < 0.05. [file Image_3.JPEG]

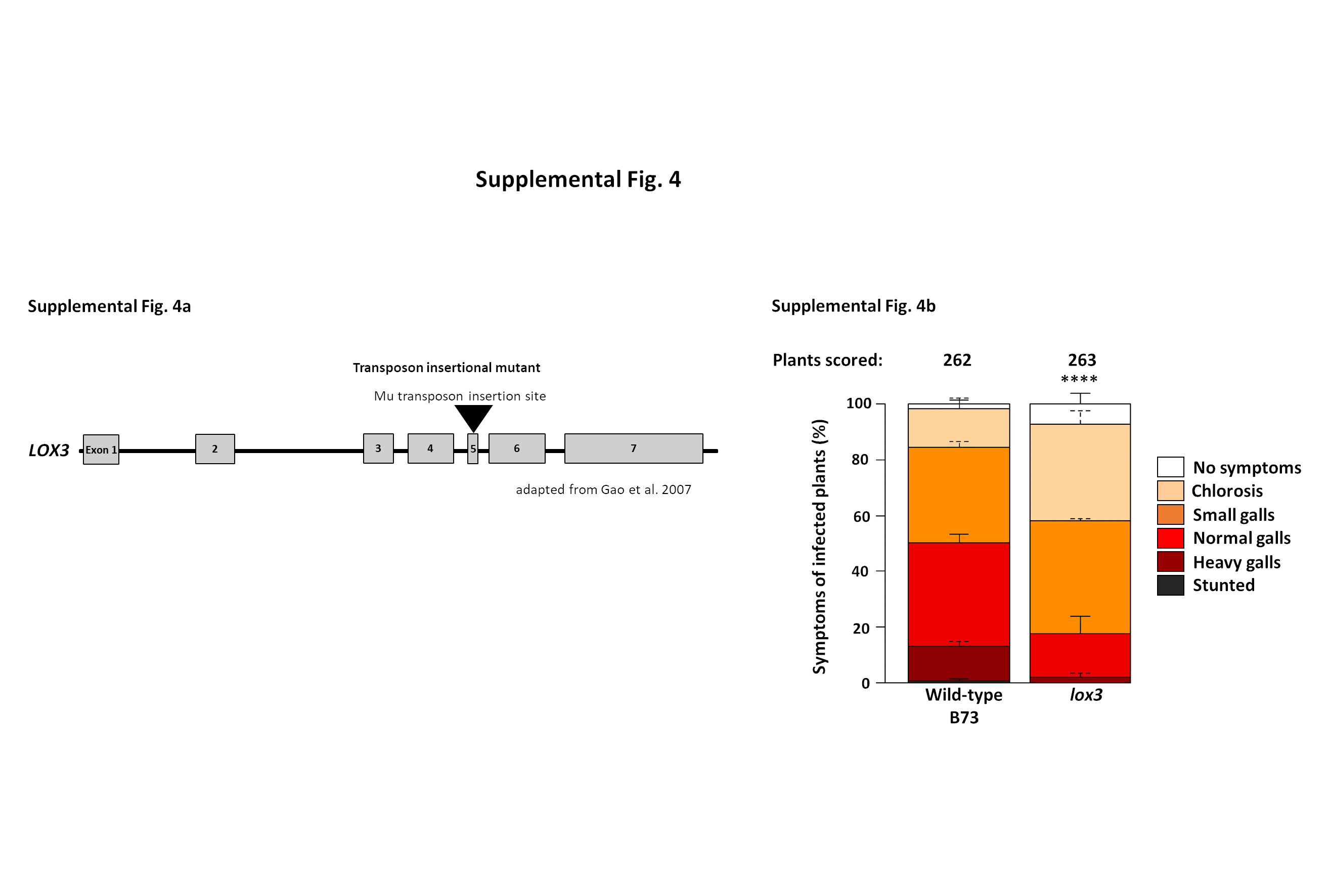

Supplement: Supplementary Figure 4 — (A) Schematic of Mu transposon insertion site in LOX3. (B) Corn smut disease rating on WT versus the lox3 mutant plants (generated via transposon insertion) as scored 8 dpi. Standard errors of the means of relative counts from three replicates are displayed. Every second error bar is dotted to facilitate the discrimination of overlapping bars. P-values were calculated by Fisher’s exact test. Multiple testing correction was done by the Benjamini-Hochberg algorithm. Asterisks indicate significant differences as compared with WT at the level of P < 0.0001. [file Image_4.JPEG]
